# Integrated analysis of circulating cell free nucleic acids for cancer genotyping and immune phenotyping of tumor microenvironment

**DOI:** 10.3389/fgene.2023.1138625

**Published:** 2023-04-06

**Authors:** Muskan Chaddha, Hemlata Rai, Ritu Gupta, Deepshi Thakral

**Affiliations:** Laboratory Oncology Unit, Dr. BRA IRCH, All India Institute of Medical Sciences, New Delhi, India

**Keywords:** cell free nucleic acids, ccfNA, coding RNA, messenger RNA, ctDNA, tumor, immune profiling

## Abstract

The circulating cell-free nucleic acids (ccfNAs) consist of a heterogenous cocktail of both single (ssNA) and double-stranded (dsNA) nucleic acids. These ccfNAs are secreted into the blood circulation by both healthy and malignant cells *via* various mechanisms including apoptosis, necrosis, and active secretion. The major source of ccfNAs are the cells of hematopoietic system under healthy conditions. These ccfNAs include fragmented circulating cell free DNA (ccfDNA), coding or messenger RNA (mRNA), long non-coding RNA (lncRNA), microRNA (miRNA), and mitochondrial DNA/RNA (mtDNA and mtRNA), that serve as prospective biomarkers in assessment of various clinical conditions. For, e.g., free fetal DNA and RNA migrate into the maternal plasma, whereas circulating tumor DNA (ctDNA) has clinical relevance in diagnostic, prognostic, therapeutic targeting, and disease progression monitoring to improve precision medicine in cancer. The epigenetic modifications of ccfDNA as well as circulating cell-free RNA (ccfRNA) such as miRNA and lncRNA show disease-related variations and hold potential as epigenetic biomarkers. The messenger RNA present in the circulation or the circulating cell free mRNA (ccf-mRNA) and long non-coding RNA (ccf-lncRNA) have gradually become substantial in liquid biopsy by acting as effective biomarkers to assess various aspects of disease diagnosis and prognosis. Conversely, the simultaneous characterization of coding and non-coding RNAs in human biofluids still poses a significant hurdle. Moreover, a comprehensive assessment of ccfRNA that may reflect the tumor microenvironment is being explored. In this review, we focus on the novel approaches for exploring ccfDNA and ccfRNAs, specifically ccf-mRNA as biomarkers in clinical diagnosis and prognosis of cancer. Integrating the detection of circulating tumor DNA (ctDNA) for cancer genotyping in conjunction with ccfRNA both quantitatively and qualitatively, may potentially hold immense promise towards precision medicine. The current challenges and future directions in deciphering the complexity of cancer networks based on the dynamic state of ccfNAs will be discussed.

## 1 Introduction

The discovery of cell-free nucleic acids in the body fluids by Mandel and Metais (Mandel, 1948) opened avenues for reliable non-invasive biomarkers only three decades later in patients with cancer (Leon et al., 1977). A vast number of studies focused on the detection of genetic aberrations rely on the isolation and characterization of circulating tumor DNA (ctDNA). The context of analysis of circulating cell-free DNA (ccfDNA) is often related to the non-invasive detection of mutations that may be susceptible to chemo-resistance, therapeutic and disease monitoring in cancer patients (Mader and Pantel, 2017; Pantel and Alix-Panabières, 2019; Thakral et al., 2020a). The ccfDNA has varied applications to evaluate different biological traits, for example, size fragment patterns, methylation status and complications during hematopoietic cell transplantation (Cheng et al., 2022), which has advanced the clinical utility of liquid biopsy in cancer patients. Albeit, the methylation status and genetic alterations harbored in ccfDNA are less dynamic and therefore, provide restricted information on tissue homeostasis and disruption.

**FIGURE 1:**
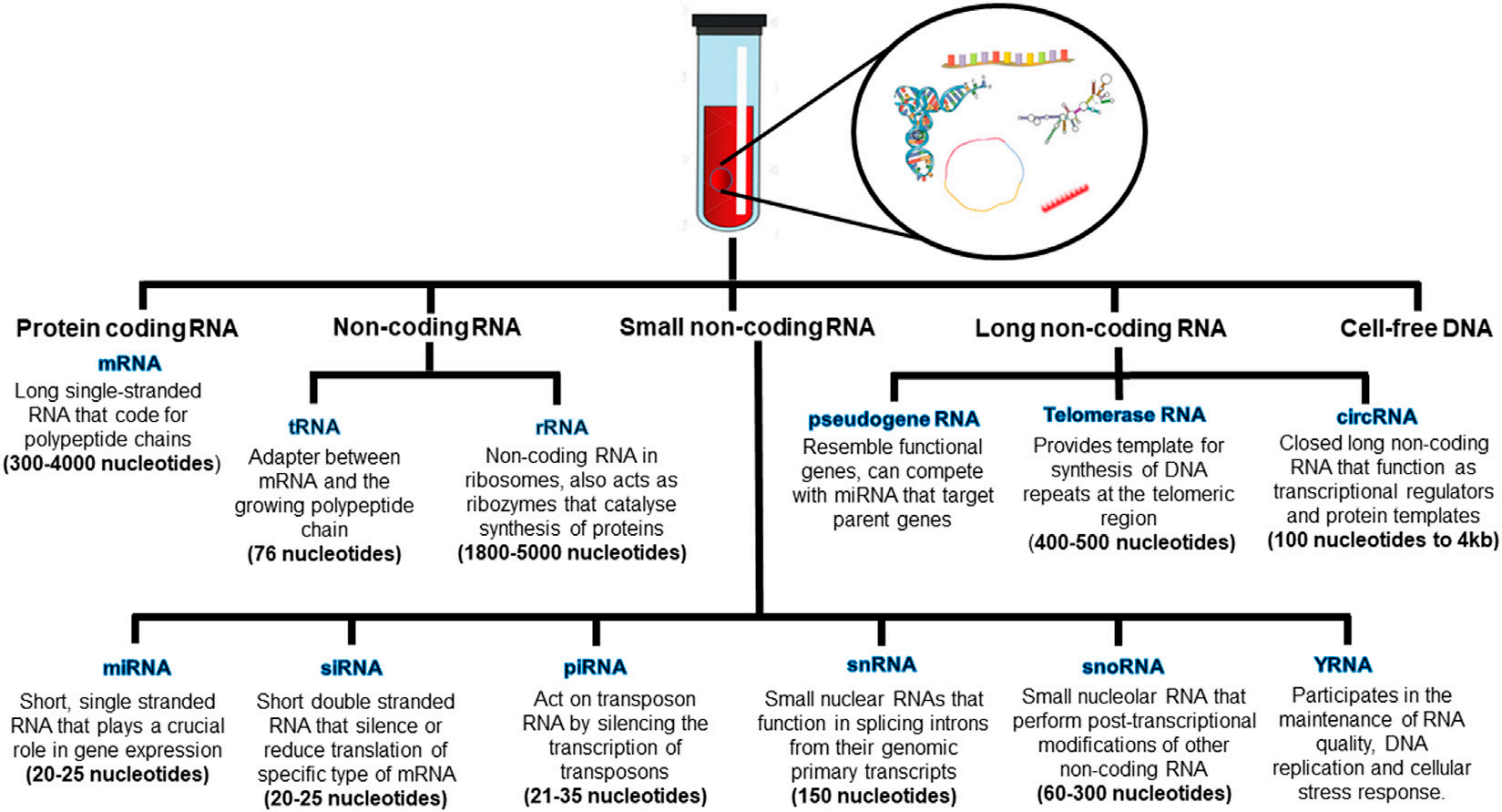
The composition of different cell-free nucleic acids and their biological function.

The heterogeneity of circulating cell free RNA (ccfRNA) further expands the battery of non-invasive biomarkers in reflecting the origin and state of the disease. Unlike the messenger or coding RNA, non-coding RNAs (micro RNA and long non-coding RNA) have been studied extensively in multiple diseases but the functionality of non-coding RNAs is still not clearly validated (Figure 1). In contrast, the circulating cell-free mRNA (ccf-mRNA) transcriptome is a direct reflection of both genetic information as well as the tissue of origin and its physiology. Several studies have reported ccf-mRNA transcripts that encode functional information of various sources including fetal development, liver, brain, and immune system (Gomez-Lopez et al., 2019).

Evidence based information indicates that the source of ccfDNA lies in the hematopoietic cells present in a healthy individual (Lui et al., 2002; Sun et al., 2015). Longitudinal studies carried out in patients who underwent transplantation and ablation of bone marrow exhibited that ccf-mRNA holds information to the temporal transcriptional activity taking place inside the bone marrow, through a non-invasive approach (Ibarra et al., 2020). Growth factors therapeutics have been used to stimulate the cell lineages in the bone marrow and the resulting data suggested that ccf-mRNA mirrors the ongoing transcriptional activity that is lineage-specific. The study concluded that ccf-mRNA delineates the transcripts of both hematopoietic and non-hematopoietic origin. About 85% of the hematopoietic transcripts were obtained from the circulating and bone marrow resident cells (Ibarra et al., 2020). Additionally, approximately 29% of the transcripts had megakaryocytic origin, ∼28% had lymphocytic origin that lies within the range of 18%–30%, 12.8% had granulocytic origin (range 6%–16%), 3% had neutrophil progenitor origin (range 0.2%–3.7%) and about 11% had erythrocytic origin (range 8%–14%). Whole blood transcriptome encompassed lymphocytic (∼69% on average) and granulocytic (∼22% on average) transcripts, along with a small fraction with erythrocytic (∼7%) origin. Profiling of ccfDNA and ccfRNA can be maneuvered to improve the existing therapeutic management of diseases of bone marrow and replace the requirement of invasive bone marrow tests. In this review, we describe the approaches of integrating the detection of ctDNA for cancer genotyping in conjunction with ccfRNA-based phenotyping including tumor immune microenvironment and current challenges to achieve them.

## 2 Circulating cell-free DNA

The fraction of circulating cell-free DNA derived from malignant cells is termed as circulating tumor DNA (ctDNA) that constitutes less than 1% of the total ccfDNA (Bettegowda et al., 2014; Kustanovich et al., 2019). These ctDNA fragments reflect the complexity and variety of the entire tumor as they cumulate from various tumor sites into circulation (Bronkhorst et al., 2019). Additionally, ccfDNA is well-suited for serial monitoring of the disease because of its easy accessibility (Schwarzenbach et al., 2011). As it correlates with disease progression, ctDNA could serve as a prognostic marker as well (Schwarz et al., 2009). Patients with various cancers (including lung, kidney, prostate, ovarian cancer, leukemia and others) had considerably higher levels of ccfDNA in their peripheral blood (∼5,000 ng/mL) as compared to healthy donors (∼13 ng/mL) (Sorenson et al., 1994; Vasioukhin et al., 1994). Additionally, between 66% and 90% of patients experienced a decline in their ccfDNA levels after radiation, and this decline was associated with both pain relief and a reduction in tumor size. On the other hand, chronically high ccfDNA levels were linked to treatment resistance, bad prognosis and advanced metastatic illness (Leon et al., 1977). Also, clinical utility of ctDNA in the metastatic setting include monitoring tumor evolution, mechanisms of treatment resistance, and guidance to switch anticancer therapies (Vellanki et al., 2023).

Despite the fact that bone marrow biopsy is a gold standard for the detection of oncogenic mutations, a study conducted on *N-RAS* mutation proposed that bone marrow aspiration does not consistently contain the diverse population of malignant clones that are implicated in the disease (Vasioukhin et al., 1994). The clinical utility of ctDNA in various hematological malignancies and other cancers had been described by our group and others earlier (Thakral et al., 2020b). It is also becoming evident that a strong correlation exists between tumor mutation burden (TMB) and the effectiveness of targeted therapy. Consequently, ctDNA is becoming a clinically advantageous substitute for biopsy for evaluating TMB (Fancello et al., 2019) and has been recommended as companion diagnostics by both Food and Drug Administration (FDA) and European Medicines Agency (EMA). The COBAS Epidermal Growth Factor Receptor (EGFR) mutation assay (Non-small lung cancer), therascreen PIK3CA RGQ PCR Kit, BRACAnalysis (Breast and Ovarian cancer) and methylation profile of SEPT9 gene (Colorectal cancer) have been approved for ctDNA-based companion diagnostic assays and several assays are also being developed for use with immuno-oncology-based therapies (Yan et al., 2021).

## 3 Circulating cell-free RNA (ccfRNA)

The circulating cell free RNA serves as a potential non-invasive biomarker whose clinical applications have not been thoroughly explored. The ccfRNA is released passively from cells undergoing processes like necrosis and apoptosis or through active secretion of membrane bound microvesicle shedding, and exosome signaling. Various types of ccfRNAs have been found in the circulation that comprises of coding, non-coding, small non-coding and long non-coding RNAs (Zhao et al., 2019) (Figure 1 **)**. The ccfRNAs isolated from body fluids (plasma and serum) have been used as biomarkers for diagnosis, early detection and provide insights on the recurrence, prediction of survivability and cancer monitoring and have been widely used in cancers of breast, prostate, pancreas, colon, thyroid and skin (Barbosa and Milas, 2008; Condrat et al., 2020). Moreover, ccfRNA measurements reflect tissue-specific changes in gene expression, intercellular signaling, and the degree of cell death occurring within different tissues because of its diverse origins.

The non-coding RNA are categorized based on their length or functionality. Depending on length, the non-coding RNA can be small ranging from 18–200 nucleotides or long that are greater than 200 nucleotides. The housekeeping ncRNA include tRNA and rRNA, whereas the regulatory ncRNA include microRNA (miRNA), small nuclear RNA (snRNA) and piwi-interacting RNA (piRNA). The small non-coding RNA have been studied extensively and are known to play diverse roles in gene regulation, RNA interference, modification or formation of spliceosome (Dozmorov et al., 2013). Moreover, the small ncRNA had been utilized as a potential biomarker for neurodegenerative diseases and may indicate the disease state based on the cell type (Tsuiji et al., 2013).

The long non-coding RNA (lncRNA) has also gathered attention of cancer researchers in the last decades (Roshani et al., 2022). The lncRNA can be derived from exosomes and has been known to play a role in metastasis, therapy resistance, tumor growth, and angiogenesis. Moreover, the lncRNA can reprogram the tumor microenvironment to promote cancer development and its progression. These exosome derived lncRNA such as lncRNA H19 have been associated with gastric cancer and can indicate patient survival. Therefore, the lncRNA can serve as promising targets for disease diagnosis and prognosis (Hashad et al., 2016).

### 3.1 Micro RNA (miRNA)

miRNA are the short non-coding RNA that are extensively studied as biomarkers for different diseases. These are ∼20–25 nucleotide long oligonucleotides and are vital for regulating the post-transcriptional expression of genes in association with mRNAs. Studies conducted by Wang et al. (2022a) reported the role of miRNA in modulation of communication between cancer and dendritic cells. miRNA have a crucial role to play in cell growth, maturation, apoptosis and proliferation, therefore when dysregulated they can act as oncogenes based on their downstream targets and drive the process of cancer development (Cheung et al., 2019). miRNA from blood fluids aid in detecting several cancers, monitoring their status and predicting their prognosis (Weber et al., 2010). Several studies have shown extracellular miRNAs in non-blood fluids like saliva, seminal fluid, breast milk, cerebrospinal fluid, tear gland secretions, aqueous and vitreous humor of the eye (Park et al., 2007), which are also associated with different types of cancer (Schwarzenbach et al., 2014). miRNA play an important role in almost every physiological as well as pathological aspect of biology and this is validated by an overwhelming sum of data that associated the dysfunctional expression of miRNA to cancer.

### 3.2 Messenger RNA (mRNA)

**FIGURE 2:**
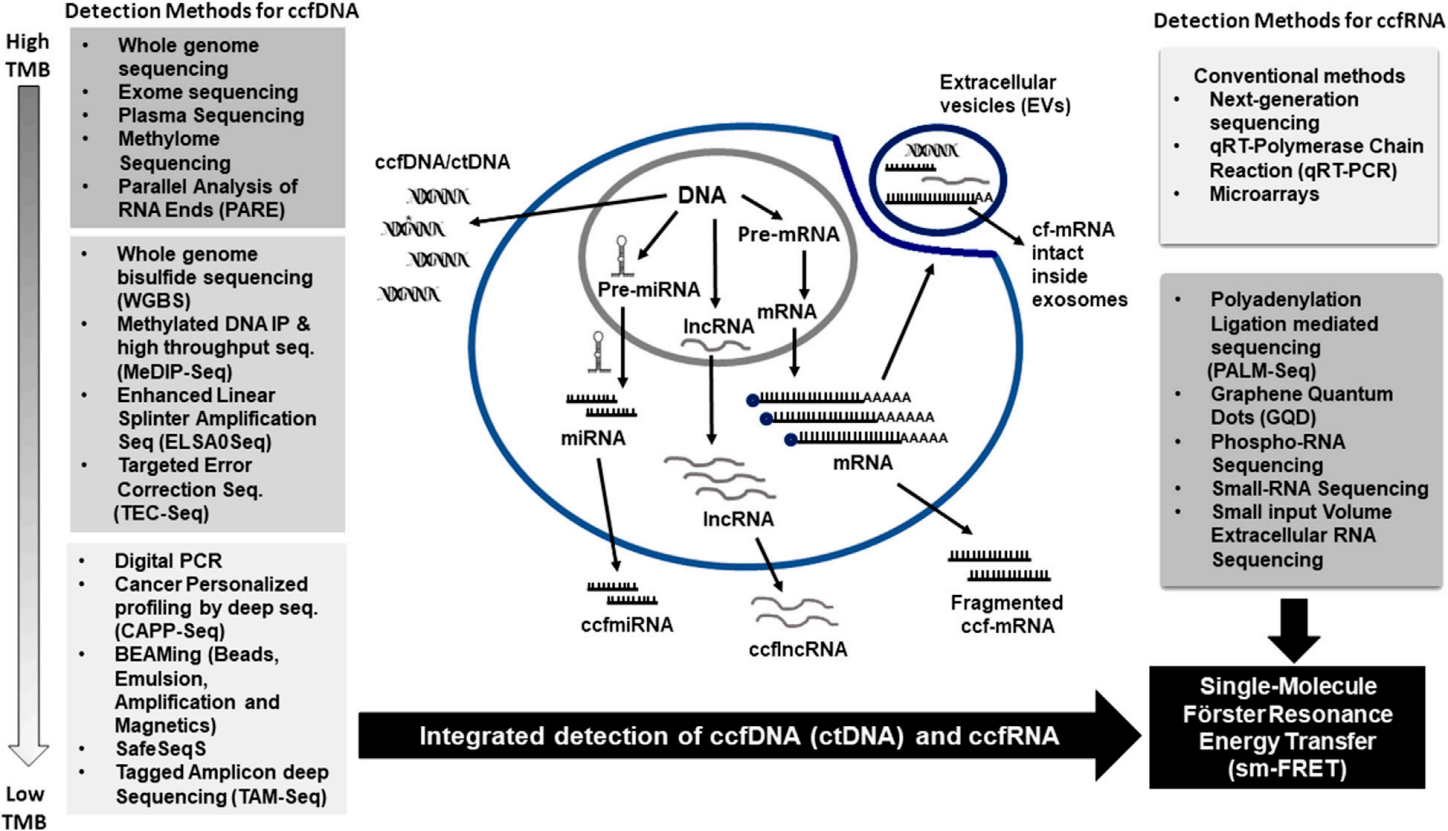
Technological advances in the detection methods for circulating cell-free Nucleic Acids (ccfNA). Circulating cell-free DNA (ccfDNA) is released from cells into the circulation by various mechanisms. High tumor burden results in more circulating tumor DNA (ctDNA) released in circulation, whereas low tumor burden correlates with lower ccfDNA that requires more sensitive detection techniques. Similarly, circulating cell-free RNA (ccfRNA) can be detected by several sensitive detection methods as indicated. Single-molecule Förster Resonance Energy Transfer (sm-FRET) can be employed for the detection of both ccfRNA and ccfDNA in circulation.

As opposed to long non-coding RNA (RNA molecules that are not translated into proteins), mRNAs are the protein coding regions that encompass the information produced as a result of DNA transcription (Figure 2). These mRNAs travel into the cytoplasm of the cell and are then shed into the blood circulation as a result of cellular secretion or lysis. The ccf-mRNA are prone to degradation and therefore it is believed that they are packaged to escape the effect of RNases in the circulation (Pös et al., 2018). However, the mechanisms involved behind such strategic packaging of ccfRNA still remain unknown. The analysis of ccf-mRNA provides a potential marker for sensitive detection of transcriptional gene expression. However, because of the presence of high RNase activity in blood, the long mRNA molecules are fragmented and can pose a significant challenge for the identification of biomarkers. Therefore, this remains a major reason for the lack of reliable ccf-mRNA biomarker candidates till date (Larson et al., 2021).

## 4 Exosomal RNA

The quest for exploring ccfRNA [both coding (mRNA) and non-coding (miRNA, lncRNA)] for their value as biomarkers in monitoring cancer, cardiovascular disorder, neurodegenerative disorder, and infection is ongoing. Various RNA species including mRNA and miRNA are released either in bound form with lipoproteins or inside exosomes as a means of cell-to-cell communication (Valadi et al., 2007). Exosomes are minute vesicles measuring a few nanometers in diameter that are released through an endocytic pathway in almost all mammalian cells. The surrounding membrane of exosomes is similar to the cellular membrane as it is also composed of the lipid bilayer that encapsulates a variety of nucleic acids (microRNA, mRNA, DNA), lipids, and cellular proteins (Haque and Vaiselbuh, 2020).

The exosomes contain a large number of mRNA transcripts and other RNA species such as miRNA and long non-coding RNA that are protected against degradation by RNases in blood circulation (Enderle et al., 2015) (Figure 2). The exosomal mRNA is crucial for regulating several biological processes and is selectively taken up by the exosomes. They act as critical mediators of intercellular communication (Wang et al., 2022a). Exosomes ferry mRNA and non-coding RNA and regulate pathogenesis and progression of various diseases. The extracellular vesicles are also present in high number in plasma from disease-free individuals that also stipulate their role apart from disease pathology (Bongiovanni et al., 2021).

The mRNA inside exosomes can potentially act as biomarkers as they are prevented from degradation by RNases in the circulation and can also remain stable at 4°C (El Fekih et al., 2021). The stability of these RNA species inside the exosomes facilitates their isolation from biological fluids. They can be regarded as significant indicators of cancer as tumor cells are known to change the expression levels of normal mRNA. The mRNA in exosomes has also been scientifically explored for their application in the evaluation of drug resistance that serves as a major barrier in cancer treatment. For instance, the levels of exosomal mRNA correlated with the intensity of temozolomide resistance in glioblastoma multiforme (Shao et al., 2015).

Similar to exosomal mRNA, miRNA can also be potentially treated as diagnostic and predictive biomarkers for diseases to reflect the internal environment of the cells along with their physiological and pathological conditions (Lu et al., 2005). miRNA in exosomes form the largest proportion of RNA and remain highly stable (for up to 5 years at −20°C) and also retain stability on multiple freeze-thaw cycles (Weber et al., 2010). Exosomes-derived miRNA released from cancer cells have been postulated to affect the functionality of healthy cells through miRNA involvement and therefore can be explored as a driving factor for cancer metastasis. The role of exosomal miRNA in colorectal cancer cells has been shown to promote the conversion of macrophages into M2 phenotype thereby supporting cell proliferation and tissue healing. Conclusively, nucleic acids inside exosomes remain relatively stable and can be used as diagnostic, prognostic and predictive biomarkers for cancer and other diseases.

## 5 Role of circulating cell free-mRNA in cancer

The extent to which ccfRNA may reflect human physiologic and disease state still remains to be elucidated. Approximately 19,000 unique transcripts of mRNA were detected in circulation among which, 65–75 percent of these coding transcripts represent the most significant proportion of circulating RNA that remains consistent with different clinical categories (Sayeed et al., 2020). Based on studies conducted by Sayeed et al. (2020), the coding transcripts in the circulation do not differ greatly from one person to another. Therefore, the circulating transcriptome is highly conserved thereby providing a reliable expression analysis.

Recent developments in the field of RNA sequencing have allowed better and efficient transcriptome analysis in plasma to facilitate the discovery and application of novel diagnostic, prognostic, and therapeutic biomarkers to detect and monitor the disease. Next-generation sequencing revealed that ccf-mRNA is enriched in the transcripts from bone marrow when compared with the transcripts from circulating cells (Ibarra et al., 2020). To support the information, longitudinal studies done after bone marrow ablation and hematopoietic stem cell transplantation in patients with multiple myeloma and acute myeloid leukemia indicated that the levels of ccf-mRNA mirrored the transcriptional state in bone marrow resident hematopoietic lineages at the time of bone marrow reconstitution. The dynamic functional changes in the hematopoietic lineages over a course of time is also associated with the cellular activity. The circulating transcriptome in the form of mRNA signifies the application of cell free transcriptome in detecting origin of tumor tissue, cancer subtype and tumor microenvironment in pathologies of bone marrow and solid cancers in a non-invasive manner.

### 5.1 Diagnostic utility of ccf-mRNA in cancer

The data from fewer studies conducted on mRNA transcripts indicated their presence as typically fragmented in the blood circulation. Studies have been conducted on the transcripts of human telomerase reverse transcriptase (hTR and hTERT), the two constituents of telomerase enzyme that are found in serum samples of breast cancer patients as potential biomarkers for disease prognosis (Chen et al., 2000). These two mRNA transcripts have also been linked to thyroid cancer and advanced melanoma and therefore these can be used as biomarkers for performing efficient diagnosis of such malignancies (Novakovic et al., 2004). Upregulation of these transcripts had also been reported in prostate, breast, gastric cancers and hepatocellular carcinoma (Kirkpatrick et al., 2003; Nakayama et al., 1998).

The thyroid stimulating hormone receptor (TSHR) mRNA could detect the presence of thyroid nodules and was utilized for predicting the rate of recurrence in thyroid cancer (Chinnappa et al., 2004). The expression of survivin relates to tumor size, nodal status, Estrogen receptor, Human Epidermal Growth factor 2 (HER2) and Progesterone receptor status along with invasion of blood vessels and clinical stage in breast cancer. These mRNA transcripts were present in high abundance in cancer as well as patients showing relapse (Yie et al., 2006). The mRNA from cyclin D1 (CCND1) and thymidylate synthase (TYMS) served as reliable predictive biomarkers in breast cancer. Subsequently the expression of CCND1 correlated to the poor outcome and resistance to tamoxifen. The combinatorial expression of CCND1 and TYMS was linked to reduced response to therapy (García et al., 2008).

Similarly, the overexpression of EGFR mRNA in circulation (isolated from peripheral blood) was linked to non-small cell lung cancer, pancreatic cancer and colon cancer (Tasdemir et al., 2019). Further analysis on the mRNA transcripts from extracellular vesicles as well as outside these vesicles indicated that the transcripts were released from solid organs such as liver and were shown to be upregulated in patients with hepatocellular carcinoma (Sayeed et al., 2020). The proportion of LINE-1 mRNA increased in the plasma-derived ccf-mRNA of the patients suffering from colorectal cancer (Filipenko et al., 2022) as summarized in Table 1.

**TABLE 1:** Diagnostic, prognostic and therapeutic utility of circulating cell free mRNA in cancer.

**Study Focus**	**Patient Cohort**	**Sample Type**	**Gene Target**	**Major Findings**	**Clinical significance**	**Reference**
hTERT mRNA and EFGR mRNA in serum as biomarker for lung cancer	112 patients (98 NSCLC, 5 SCLC, 1 LCLC and 8 benign tumor) 80 controls	Serum	hTERT and EGFR	**EGFR-** cell survival, cell proliferation, invasion, angiogenesis, metastasis, dissemination of cancer cells **hTERT**- size and number of tumors, tumor marker progression	Diagnostic markers	Miura et al. (2006)
Role of ccf mRNA in plasma as tumor marker for gastric cancer; primary and recurrent	52 gastric cancer patients (40 preopretaive + 12 postoperative) 20 healthy controls	Plasma	hTERT and MUC1	**hTERT and MUC1-** Over expression in gastric cancer as released from neovessels in cancer tissues. Tumor derived mRNA released from tumor tissues detected in circulation in the absence of circulating tumor cells.	Diagnostic markers for primary gastric cancer at an early stage. Diagnostic markers for clinically occult recurrences after gastrectomy.	Tani et al. (2007)
Role of mRNA in breast cancer patients and their clinical outcome	129 patients	Plasma	Cyclin D1 and thymidylate synthase (TYMS/ TS)	**TYMS mRNA-** poor prognosis, negative progesterone receptor, advanced stage in colon cancer **Cyclin D1 mRNA**- Vascular evasion, oncogenic transformation, no response to therapy, shorter overall survival	TYMS mRNA-prognostic biomarker for advanced stages. Cyclin D1- prognostic biomarker for overall survival and therapy resistance.	García et al. (2008)
Extracellular tumor related mRNA in lymphoma and survival	42 diffuse large B-cell lymphomas (DLBCL) 25 follicular lymphoma (FL) 16 Hodgkin's lymphoma (HL)	Plasma	MYC, CCND2 and BCL2-unfavourable biomarkers BCL6, LMO2 and FN1-favourable biomarkers	**LMO2 (LIM Domain Only 2)**-prolonged survival**MYC, CCND2 (cyclin D2) and BCL2 (B-cell lymphoma 2)**-poor prognosis, short survival, poor outcome and partial response (MYC mRNA), treatment resistance (MYC or BCl2) **BCL6-**better survival, favorable outcome,absence of unfavorable biomarkers	MYC, CCND2, BCL2, and BCL6-prognostic biomarkers MYC and BCL2-therapy resistance	Garcia et al. (2009)
hTERT mRNA as a diagnostic biomarker for hepatoma	638 patients (303 HCC, 89 chronic hepatitis, and 45 liver cirrhosis) 201 controls	Serum	hTERT	**hTERT mRNA** tumor size, tumor differentiation, superior to AFP (α-feto protein), AFP-L3 and DCP (des-γ-carboxy prothrombin) in diagnosis recurrence of HCC at early stage	Diagnostic biomarker	Miura et al. (2010)
hTERT mRNA as prognostic biomarker for prostate cancer	105 patients 68 healthy volunteers	Plasma	hTERT	**hTERT mRNA-** discriminates clinically localized and locally advanced disease, recurrence of prostate cancer, poor prognosis, tumor stage, vascular and perineural invasion, tumor size, metastasis	Diagnostic biomarker, MRD detection, prognostic biomarker for recurrence	March-Villalba et al. (2012)
BRCA1 and TS mRNA as biomarker for chemotherapy against gastric cancer	150 patients	Plasma	BReast CAncer gene (BRCA1) and Thymidylate synthase (TS)	**BRCA1 mRNA-** biomarker for selecting chemotherapy during disease progression monitoring of neoadjuvant and adjuvant therapy chemosensitivity **TS-** pemetrexed-based chemotherapy BRCA1- Docetaxel therapy	Therapeutic biomarkers	Shen et al. (2014)
CfmRNA and miRNA as biomarkers for prostate cancer	102 untreated Prostate cancer 50 disease free controls	Plasma	OR51E2, SIM2 mRNA miR-200c and miR-200b	**SIM2 and OR51E2-** prostate cancer development and progression, target for immunotherapy, better diagnosis than PSA test when combined with miR-200c and miR-200b, cancer diagnosis with PSA≤4 ng/mL **miR-200c-** bone metastasis, PSA level > 10 ng/µL, Bilateral tumor	Prognostic biomarkers	Souza et al. (2017)
Role of cfmRNA in predicting high risk prostate cancer	60 prostate cancer	Plasma	AMACR, BCL2, NKX3-1, GOLM1, OR51E2, PCA3, SIM2 and TRPM8	**AMACR, GOLM1, TRPM8 and NKX3-1-** overexpressed, associated with disease, aggressiveness, extracapsular extension, tumor stage, vesicular seminal invasion **GOLM1, TRPM8 and NKX3-1-** identification of high-risk prostate cancer with high sensitivity and specificity **NKX3-1-** Prostate cell differentiation, Prostate cancer initiation, (loss of function), high gradeprostate cancer (overexpression) **TRPM8-** cell migration and tumor progression	Diagnostic biomarkers	De Souza et al. (2020)
Role of GRP78 as biomarker for endometrial cancer	32 patients with endometrial cancer 20 healthy controls	Plasma	GRP78	**GRP78-** overexpressed in patients with endometrial cancer	Diagnostic marker	Aynekin et al. (2022)

### 5.2 Prognostic value of ccf-mRNA in cancer

Increased levels of metastatin mRNA in the serum correlated with poor survival rate and metastasis to lymph nodes in breast cancer (El-Abd et al., 2008). The changes in the expression levels of carcinoembryonic antigen (CEA) mRNA, serum collagen type VI type alpha 3 chain (COL6A3) and alpha-1,4 acetylglucosaminyltransferase (α4GnT) mRNA were closely linked to the cancer of pancreas and about 33% of the patients with pancreatic carcinoma and 22% patients with gastric cancer were positive for CEA mRNA in the peripheral blood samples (Sayeed et al., 2020). High expression of free circulating RNA of Ribonucleotide Reductase Catalytic Subunit M1 (RRM1) gene was significantly associated with increased risk of grade 3 Head and Neck cancer (Mlak et al., 2018).

The first study in ovarian cancer demonstrated circulating Metastasis-associated in colon cancer 1 (MACC1) and S100 calcium-binding protein A4 (S100A4) transcripts as potential liquid biopsy markers (Link et al., 2019). Both MACC1 and S100A4 are implicated in tumor progression and metastasis. The clinical relevance of serum circulating ccf-mRNA of MACC1 and S100A4 genes was shown by systematic tracking in due course of surgery and chemotherapy. High levels of MACC1 or S100A4 ccf-mRNA correlated with advanced FIGO stage (*p* = 0.042; *p* = 0.008), at diagnosis predicting suboptimal debulking surgery and indicated shorter progression-free survival (PFS; *p* = 0.003; *p* = 0.001) and overall survival (OS; *p* = 0.001; *p* = 0.002).

The ccf-mRNA has been employed for risk assessment of prostate cancer. Transcripts of *AMACR*, *GOLM1*, *TRPM8* and *NKX3-1* genes were overexpressed and were closely associated with cancer aggressiveness, extracapsular extension and vesicular seminal invasion (De Souza et al., 2020). A combination of genes circulating mRNA signature (*GOLM1*, *NKX3-1* and *TRPM8*) was able to identify high-risk prostate cancer cases (85% of sensitivity and 58% of specificity). This ccf-mRNA signature has emerged as a superior marker with a better overall performance compared with the classical biopsy Gleason score and prostate-specific antigen. Pre-transplant measurable residual disease status of WT1 ccf-mRNA expression has been assessed for prognostic value in peripheral blood obtained from patients with Acute Myeloid Leukemia and Myelodysplastic syndrome (MDS) (Rautenberg et al., 2021).

### 5.3 Role of cell-free transcriptome in cancer profiling and monitoring

Comprehensive non-invasive profiling of circulating cf-mRNA by next-generation sequencing revealed dysregulated genes in the ccf-mRNA of Alzheimer disease (AD) patients, which were enriched in AD-associated biological processes (Toden et al., 2020). Albeit, the first transcriptome-wide characterization of ccfRNA in cancer (stage III breast and lung [*n* = 46 + 30]) and non-cancer (*n* = 89) participants from the Circulating Cell-free Genome Atlas (NCT02889978) was reported by Larson et al. (Larson et al., 2021). Of 57,820 annotated genes, 39,564 (68%) were unique in cancer samples. This study identified cancer-specific genes and their tissue of origin, defined as “dark channel biomarker” (DCB) genes, that are recurrently detected in individuals with cancer. The promising indicator were the DCB levels in plasma that correlated with tumor shedding rate and RNA expression in matched tissue. These findings were of great utility in patients with low levels of circulating tumor DNA wherein the high expression of DCB (ccf-mRNA) could enhance cancer detection.

Notable differences were reported in the plasma transcriptomic profile between low disease burden compared to advanced melanoma patients (Ita et al., 2021). Several genes were significantly over-expressed in patients with a low disease burden or therapeutic response (*BCL2L1*, *CXCL9*, *IDO1*, *IL13*, *MIF*, *MYD88* and *TLR4* *p* ≤ 0.001, versus high disease burden). In patients with therapeutic response relative to baseline assessment, a statistically significant increase in *BCL2L1*, *CCL4*, *CCL5*, *CXCL9*, *GZMB* and *TNFSF10* genes was noted. Moreover, in stage IV melanoma patients with brain metastases, *CCL18*, *CCR1*, *CCR4*, *CD274*, *CSF2*, *EGF*, and *PTGS2* genes were significantly over-expressed (*p* < 0.001, versus patients without melanoma brain metastasis).

In another study, ccf-mRNA facilitated the minimally invasive pan-tumor monitoring of melanoma therapy that was not dependent on the mutational genotype of the patient (Albrecht et al., 2022). High diagnostic accuracy was shown based on ccf-mRNA expression of *KPNA2*, *DTL*, *BACE2* and *DTYMK* genes between melanoma patients’ and healthy donors’ plasma (AUC >86%, *p* < 0.0001). Furthermore, ccf-mRNA levels correlated proportionally with increasing TMB and radiological absence of disease. The cellular sources of ccfRNA were traced by comparison with transcriptomes obtained from single cells, that identified a pan-tumour origin beyond melanoma cells (including endothelial, cancer-associated fibroblasts, macrophages and B cells). The genes with low baseline ccfRNA levels were associated with a prominently longer progression-free survival (PFS) and overall survival (OS). An increase in the ccfRNA levels of *KPNA2* and *DTYMK* at the time of therapy predicted shorter PFS. Supportively, the levels of ccfRNA substantially increased after therapy in non-responders when correlated against the responders, independent of therapy and mutational subtypes.

Plasma ccfRNA expression levels were quantified by a tagmentation-based library sequencing in primary NSCLCs (Seneviratne et al., 2022). The comparison of ccfRNA expression levels between patients and control groups revealed a total of 2,357 differentially expressed ccfRNAs enriched in 123 pathways. This study further provided a framework for developing blood-based assays for the early detection of NSCLC. Hence, cell-free RNAs circulating in plasma offer a non-invasive detection of spatial and temporal changes occurring in primary tumors since the early stages.

### 5.4 Role of cell-free transcriptome in tumor immune profiling

Immune cell types and hematopoietic tissues are the primary contributors to the cf-transcriptome cell type landscape as described in the previous sections. The tumors can be phenotypically categorized into “hot,” “cold” or “altered” according to the rate of infiltration of CD3^+^ and CD8^+^ T-cells at the center of tumor and also alongside its margin (Immunoscore) (Galon and Bruni, 2019), expression of the checkpoint molecules (PD-1 and PD-L1) and tumor mutation burden. These parameters are also strong determinants for predicting the response of cancer to immunotherapy. An urgent need exists for the development of reliable non-invasive approaches that can appropriately assess and monitor the different molecular dysregulations that are linked to the immune and inflammatory reactions inside the blood circulation and also in inaccessible solid organs of the body.

A pre-clinical mouse model was utilized to temporally demonstrate the potential of profiling ccf-mRNA in plasma through drug-induced molecular alterations that are associated with inflammatory and immune responses. On one hand, lipopolysaccharide induced systemic immune responses and dysregulated signaling pathways were captured by cell-free mRNA-Seq whole transcriptome profiling. On the other hand, JAK inhibitor was shown to diminish these inflammatory pathways, including interferon and STAT signaling that was reflected in the ccf-mRNA (Zhuang et al., 2022). Interestingly, the ccf-mRNA harbored alterations in gene expression of liver-specific transcripts, which is otherwise inaccessible.

A noteworthy study demonstrated the differential expression of genes involved in cancer inflammation and immunity. Among patients with different glioma grades, a positive correlation between their transcriptomic profile in plasma and tumour samples was shown (Ita et al., 2021). The *BCL2L1*, *GZMB*, *HLA-A*, *IRF1*, *MYD88*, *TLR2*, and *TP53* genes were significantly over-expressed in glioma patients (*p* < 0.001, versus control). Immune-related genes *GZMB*, *HLA-A* and *BCL2L1* were significantly over-expressed in high-grade glioma patients (*p* < 0.001, versus low-grade glioma patients) and a concordance between differentially expressed genes in plasma- and glioma-derived RNA was reported.

### 5.5 Role of cell-free transcriptome in therapy resistance

A potential usability of plasma-derived exosomal RNAs was described in non-small cell lung cancer patients for the characterization of molecular phenotypes of osimertinib resistance (Alexeyenko et al., 2022). The transcriptome landscape of Osimertinib-refractory NSCLC patients was attributed to the involvement of multiple RNA species. Transcriptome profiling revealed differential expression of 128 transcripts and enrichment analysis revealed alterations in pathways related to EGFR and PI3K as well as to syndecan and glypican pathways. This longitudinal study, sampled plasma of osimertinib-treated EGFR T790M NSCLC patients that could provide biomarkers of acquired resistance to osimertinib.

### 5.6 An integrated approach of ccfDNA and ccfRNA in tumor and immune profiling

The combination of ccfDNA along with cf-miRNA has been extensively studied as biomarker for diseases such as cancer, neurological ailments, and cardiovascular diseases. However, there are fewer studies conducted on ccf-mRNA as a source of biomarker. Few mRNA transcripts like *LMNB1*, *TGF-β*, and *MCM6* from plasma samples were proposed as potential biomarkers for hepatocellular carcinoma. The MYC mRNA from serum samples of B-cell lymphoma was linked to short overall PFS and partial treatment response. However, some restrictions that limit the use of mRNA in clinical practice can be attributed to the labile nature of mRNA in circulation (Whitney et al., 2003).

The ctDNA that is released by tumor cells can give insights on specific mutations in the genes that can form a basis for targeted therapy. Various ctDNA-derived screening tests are approved and are being routinely used. ctDNA is closely linked to the tumor burden and is released exclusively by the tumor cells in the circulation (Chattopadhyay, 2020). However, the limitation associated with the use of ctDNA is that it is not released in a detectable amount in early stages that can facilitate early detection of cancer stage with fewer tumor cells (Cheung et al., 2019). Contrasting to this, ccf-mRNA is released from cancer as well as non-cancer cells, and so it is released by the cells that are not yet transformed. Such a characteristic allows early detection and diagnosis of cancer and other malignancies (Nabet et al., 2017).

The ALK, ROS1 and RET kinase fusion are crucial biomarkers that can predict the function of tyrosine kinase inhibitors (TKIs) in the non-small-cell-lung cancer (NSCLC). The following study proposed the applicability of both ccfRNA and ccfDNA for detection of fusion genes. A standard protocol facilitated the detection of fusion genes by ccfRNA and formulated analytical validity and functionality in 30 samples from 20 patients with fusion positive NSCLC. The conclusions drawn from ccfRNA-based assays were compared against the ccfDNA based assay (Guardant360 plasma NGS assay). The cumulative sensitivity of the ccfRNA based assays was 26.7% as opposed to 16.7%, for ccfDNA based assay. The analysis that was restricted to the samples obtained at chemo-naïve or progressive disease status and available for both assays showed that sensitivity of ccfRNA based assays was comparatively higher about 77.8% than ccfDNA based assay (33.3%). The identification and characterization of fusion-genes in ccfRNA correlates with the treatment response and these results support the usefulness of ccfRNA assay in diagnosing patients with insufficient tissues to channelize proper first-line treatment. Subsequently, the assay can also be used to monitor the progression of NSCLC that can be further considered for second-line of treatment (Hasegawa et al., 2021).

## 6 Advances in the platforms for detection of circulating cell free nucleic acids

The cell free nucleic acids in circulation hold great potential as minimally invasive biomarkers for diagnosis and prognosis of cancer. Both plasma and serum serve as a major source for isolation of cell free nucleic acids but plasma is the preferred choice for ccfDNA. Since the ctDNA is relatively low in concentration, especially in the early stages of cancer, therefore highly sensitive advanced detection methods for successful profiling of mutation, methylation, and fragment patterns are recommended. Various conventional and ultrasensitive technology platforms for genotyping ctDNA were described by our group and others earlier (Park et al., 2017; Thakral et al., 2020b). The sequencing of ccfDNA based on epigenetic alterations has also emerged as a promising method for the early detection of cancer by evaluation of methylation of CpG sites to understand the mechanisms of gene expression, tissue differentiation, organ development, aging, and angiogenesis (Köhler and Rodríguez-Paredes, 2020).

Further technological advancements have been witnessed towards better characterization of ccfDNA (Figure 2). Cell-free Methylated DNA Immune Precipitation and high throughput sequencing (MeDIP-Seq) is an immune-precipitation-based method that enables genome-wide methylome profiling with low input of DNA as is present in the circulation in early stages of cancer. Other advanced techniques like methylBEAMing, single-cell RRBS (Reduced Representation Bisulfite Sequencing), and Enhanced Linear Splinter Amplification-Sequencing (ELSA Seq) simplify the application of ccfDNA methylation sequencing by acting on the prerequisites and reducing the need for high input volumes of DNA and also enhancing the analytical sensitivity (Liang et al., 2021). ELSA-Seq infer details on deep methylome coverage with reduced amplification bias using single-stranded libraries with ultra-low input DNA (as low as 500 pg).

Targeted error correction (TEC-Seq) based sequencing is a recent UMI-based technique for ccfDNA detection that has been employed for detection of mutations in 58 genes involved in cancer of ovaries, breast, and lung (Phallen et al., 2017). However, the targeted sequencing is primarily dependent on the prior information of genetic mutations in respective cancers that poses a major limitation. Whereas, methylation-based and ccfDNA fragmentation-based approaches may be more informative on early cancer detection.

The lack of optimum reproducibility across different studies occurring from lack of knowledge of standard isolation methods for ccfRNA has restrained the progress in the applications and versatility of liquid biopsies. A comparative analysis of ten methods used for isolation of ccfRNA from different biofluids have provided insights on the complexity and reliability of consequent small RNA-seq profiles (Srinivasan et al., 2019). An assessment on the persistence of extracellular vesicle and ccf-mRNA profiles found in the plasma during blood sample processing and its storage has also been documented (Kim et al., 2022).

Similarly, the gene expression characterization of ccfRNA involves the use of conventional methods such as quantitative real time-polymerase chain reaction (Taqman, LNA-based, or SYBR Green), microarrays and Next-generation sequencing (Figure 2). Every method comes with its unique set of pros and cons depending on the experimental design (Bernardo et al., 2012). The mRNAs and lncRNAs in the plasma are present in fragmented form and are usually missed by standard form of small RNA-seq protocols as they are devoid of 5′phosphate or have 3′phosphate group.

Modifications have been introduced in small RNA sequencing strategy to facilitate the simultaneous detection of ccfRNA. Yao J et al. performed Thermostable group II intron reverse transcriptase sequencing (TGIRT-Seq) in combination with peak calling to concomitantly profile the different biotypes of RNA in human plasma that was pooled from different disease-free individuals. From this study, the researchers proposed that the plasma contains mRNA that has undergone substantial fragmentation derived from more than 19,000 protein-coding genes, high population of full length and mature tRNA and other structured small non-coding RNA with a minor population of mature and pre-miRNA (Yao et al., 2020). Different mRNA fragments that were observed in the plasma samples were linked to protein-binding sites with structurally stable predicted secondary structures that were also resistant to nucleases found in the plasma. Additionally, the novel repeat RNA, putatively structured intronic RNA and miRNA-sized RNA have been identified that contain potential information of evolutionary and biological significance as well as probable biomarker candidates.

A novel detection technique that determines small fragments of RNA called “Phospho-RNA-Seq” reveals that other standard small RNA-seq techniques are not sensitive enough to detect circulating mRNA and lncRNA in human plasma that can potentially serve as biomarkers. Therefore, this new method used treatment of RNA with T4-polynucleotide kinase in assessing these small circulating RNA fragments (Giraldez et al., 2019). A parallel study performed a comparison of conventional methods used for sequencing small RNA fragments by small-RNA sequencing (sRNA-seq) and sRNA-seq using T4 polynucleotide kinase (PNK). The latter also performed end-treatment of total extracellular RNA that was isolated from serum and platelet-poor EDTA, acid-citrate-phosphate and heparin plasma to understand the impact on extracellular-mRNA acquisition. When compared against the conventional sRNA-seq methods, the newly devised PNK-treated methodology provided better detection of essential extracellular mRNA reads and increased sensitivity to about 50 folds (Akat et al., 2019). The extracellular RNA bulk was found to be dominated by the hematopoietic cells and the platelets derived from liver. The serum also contained approximately 2,300 transcripts that were twice the number of transcripts isolated from plasma. The EDTA and ACD treated plasma affected the stability of extracellular mRNA and non-coding RNA ribonucleoprotein complexes.

Further modification was described by Liu et al. (2022) by development of polyadenylation ligation-mediated sequencing (PALM-Seq), which enables a simultaneous characterization of ccf-mRNA and ccf-lncRNA. In this RNA sequencing workflow, RNA is treated with T4 polynucleotide kinase to generate cell-free RNA fragments with 5′phosphate and 3′hydroxyl followed by RNaseH treatment for depletion of excess RNAs, thus achieving simultaneous quantification and characterization of ccfRNAs in biofluids, plasma and amniotic fluid.

**FIGURE 3:**
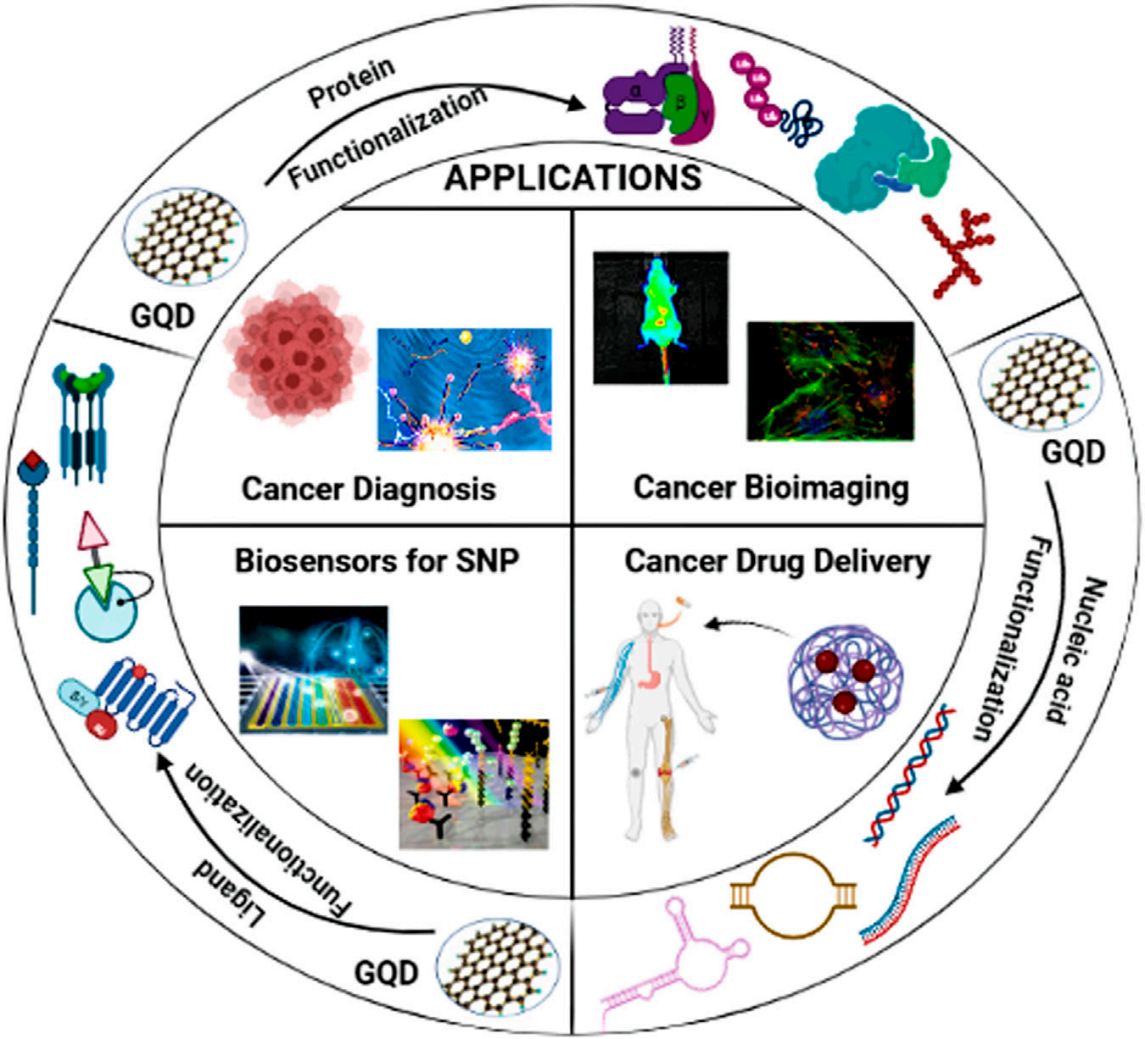
Graphene quantum dots (GQDs) and their surface biofunctionalization for different sensing platforms.

Newer methods for biomolecule detection through graphene quantum dots (GQDs) was described that could estimate the circulating nucleic acids like DNA, mRNA, miRNA, mtDNA and lncRNA in plasma and serum samples (Ratre et al., 2022). These GQDs have better sensitivity and effectiveness when collaborated with optical, chemiluminiscent and electrical biosensors to selectively identify circulating cell free nucleic acids and transform into signal-specific biomarkers (Goryacheva et al., 2018). The small size of these GQDs and properties like edge effect and quantum confinement hold the potential of developing into an effective diagnostics in bioanalysis and detection of ccfNAs (Tian et al., 2015) (Figure 3).

SILVER-Seq (Small Input Liquid Volume Extracellular RNA Sequencing) is a newly proposed method for sequencing integral as well as fragmented form of ccfRNA in as small as a droplet of volume 5-7 µl from liquid biopsy (Zhou et al., 2019). This technique has the ability to detect extracellular RNA from more than one-fourth of the human genes that also constitutes small RNAs, fragmented mRNAs and lncRNAs. The extracellular RNAs (exRNA) that were detected also comprised the ones derived from genes that displayed tissue-specific expression (e.g., brain specific exRNA). There was a prominent difference in extracellular RNA expression between the breast cancer and non-cancer donors. A moderate difference in this expression also existed in donors with or without cancer recurrence.

Short-read deep RNA-Seq has been reported to identify the mRNA, lncRNAs and circRNAs (circular RNAs) in plasma as novel biomarkers for diagnosing coronary artery disease (CAD). The circulating cell-free RNA was sequenced in patients with stable CAD and controls. The fragments from 160 of 3,986 mRNA, 10 of 164 lncRNAs and 2 of 405 novel lncRNAs were observed to be altered in patients with disease. The transcripts that were differentially elevated were enriched in mRNA that is coded by mitochondrial DNA that stipulates myocardial ischaemia and oxidative stress (Ward et al., 2022).

FRET-based intramolecular kinetic fingerprinting for direct digital counting of single molecules of nucleic acid that is also known as intramolecular single molecule recognition through equilibrium Poisson sample (iSiMREPS) is employed for a quick determination and counting of miRNA and mutant ctDNA with a very high sensitivity and specificity to detect as small as single molecule (Khanna et al., 2021) (Figure 2). This is done by using pair of fluorescent detection probes, where one probe is immobilized to the target present on the surface and the other probe molecule binds to the target transiently and reversibly to produce unique time-resolved fingerprints in the form of smFRET signals. These signals are further detected by total internal reflection fluorescence microscope. The iSiMREPS has been utilized for quantification of EGFR exon 19 deletion and miR-141 in ctDNA molecules with an extremely sensitive limit of detection of ∼1 and 3 fM, respectively and in mutant allele fractions as low as 0.0001%, thereby demonstrating its wide application in clinical diagnostics.

## 7 Discussion

**FIGURE 4:**
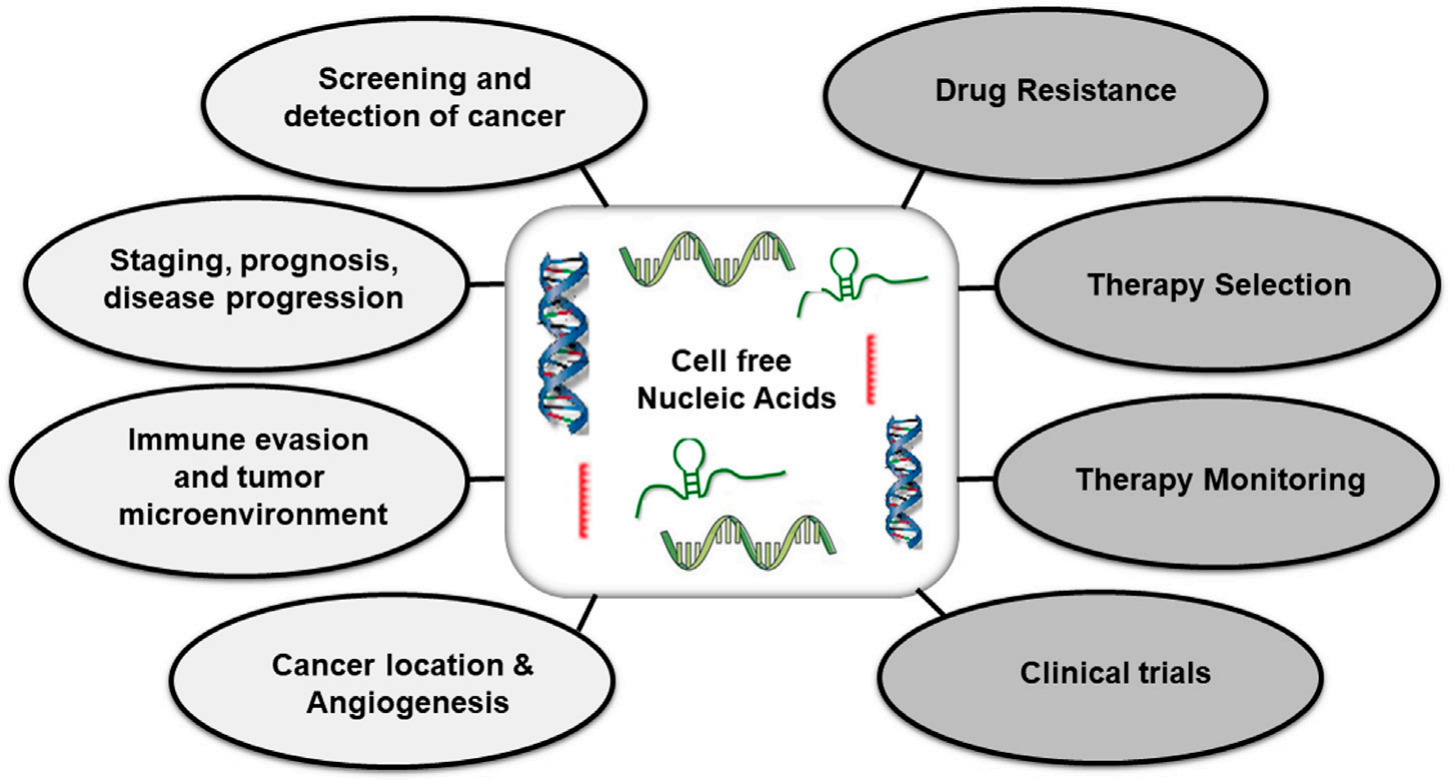
Applications of cell free nucleic acids in diagnosis, prognosis, disease monitoring (Left panel) and treatment (Right panel) of malignancies.

The ccfDNA has been employed in the prediction of transplant rejection, disease monitoring, predicting prognosis, prenatal diagnosis, relapse of disease, and clinical trials (Figure 4). Regardless of advances, ccfDNA is primarily confined to understanding the disease physiology manifested due to genetic alterations (Bianchi and Chiu, 2018). The potential of ctDNA as a prognostic biomarker has not been formally validated specially in solid tumors after definitive surgery or chemoradiation. There are few notable limitations to these studies that warrant standardization of testing. The sensitivity of ctDNA for MRD detection is influenced by sampling time, duration of follow-up, propensity of tumors for ctDNA shedding, assay characteristics and performance impact.

Circulating cell free RNA, on the other hand that has emerged alongside ccfDNA, holds the transcriptome which can be contemplated as transcript from different tissues (Koh et al., 2014). The ccf-mRNA transcriptome profiling provides information on the well-characterized tissue-specific genes that support the understanding of the biomolecules to assess disease or health. Success has been achieved with ccfRNA that has been employed to detect and characterize pregnancy-related conditions like pre-eclampsia, spontaneous pre-term birth and intrauterine growth restriction (Hannan et al., 2020). When compared to the levels of ccfDNA in circulation, the changes in the levels of ccfRNA during the period of gestation is more predictable that pave the way to a close and definitive understanding of gene expression in fetus and placenta (Ngo et al., 2018; Munchel et al., 2020).

The ccfRNA contains necessary signatures in the characterization of oncological patients that have a strong potential to be developed as markers alongside ctDNA for diagnosis and monitoring (Pös et al., 2018). This ccfRNA can also be employed in tumor characterization and the monitoring of personalized treatments in liquid biopsies obtained from cancer patients. A thorough analysis of ccfRNA can impart complementary information on the gene expression profiles as well as epigenetic changes in healthy versus tumor tissue (Geeurickx and Hendrix, 2020). The disrupted balance between the circular RNA and its linear mRNA causes aberrant expression of tumor suppressor genes and oncogenes (Dragomir and Calin, 2018). For, e.g., the analysis of ccfRNA in liquid biopsy indicated higher expression of miRNA in plasma and most of these miRNA interacted with the genes present in the PTEN-PI3K-AKT-mTOR pathway that is crucial for the development of endometrial cancer (Pardini et al., 2019). Therefore, ccf-mRNA has sufficient specificity that can be exploited to diagnose different types of cancers at different stages.

Although, the detection and study of ccfRNA is an incredible discovery but RNA is more highly prone to degradation due to the presence of several ribonucleases in the blood. It is shown that RNA is present inside apoptotic bodies, other vesicular structures, or in bound form within DNA nucleosomes to escape the denaturing action of RNases in the blood (Sisco, 2001). *In vitro* studies carried out on serum had shown that RNA present inside the apoptotic vesicles does not undergo degradation (Hasselmann et al., 2001). The levels of extracellular RNA are increased in patients with cancer and therefore its application as a potential biomarker for cancer has been confirmed (Ng et al., 2002).

The mRNA is released from the cells through apoptosis and necrosis however it is also shed from tumor tissues even at a low abundance of circulating tumor cells. As shown with circulating cell-free hTERT and MUC1 mRNA that can provide a screening tool for the early detection of gastric cancer and its relapse (Tani et al., 2007). The ccfRNA is encased inside extracellular membrane vesicles (EMV) to escape the degrading effects of RNases or they can form complexes with ribonucleoproteins that also functions to secure the RNA once released in circulation. The molecules of ribonucleic acids are packed in a selective manner based on their origin and viability (Lázaro-Ibáñez et al., 2014). RNA inside the living cells is secreted through microvesicles and exosomes, where exosomes are the smallest type of EMV and are crucial in establishing intercellular communication (Yamamoto et al., 2016). Nevertheless, several challenges need to be overcome for exploiting the combined translational potential of ccf-mRNA and ctDNA in clinical settings.

### 7.1 Technical challenges

The major challenge that persists in the applicability of liquid biopsies as definitive screening, diagnostic and prognostic tools is the issue of biological and technical reproducibility and the absence of any gold standard for sample storage, processing, and data analysis (Geeurickx and Hendrix, 2020). Apart from these, there also exists technical bias when handling samples due to external factors (age and sex) that have a substantial impact on the cell-free RNA profile (Rounge et al., 2018; Wagner et al., 2020). The presence of high technical variability in different studies also results in finding unrelated RNA signatures for the same disease and with the same type of sample. In order to mitigate this variability high-throughput screening methods, genomics, and other data-intensive disciplines can be employed to allow standardization (Freedman and Inglese, 2014).

### 7.2 Biological challenges

An efficient RNA biomarker discovery is also hindered by biological challenges that include presence of high interpersonal variability, that implies that the expression of some genes is higher in certain individuals than others. This stipulates the importance of invariable normalization to address this biological variability.

### 7.3 Challenges in bioinformatic analysis

One of the challenges encountered with biomarker identification and discovery using RNA sequencing is the interpretation and analysis of the data obtained. The bioinformatics analysis depends on data quality control, quantification of the transcript, and other downstream processes. Furthermore, the input quality also serves as a critical parameter for the quality assessment of the data. RNA sequencing is an extensively used tool for estimating gene expression, however it does not provide absolute quantification of the data like RT-qPCR methods (Cabús et al., 2022).

This limitation maybe overcome by normalization based on spike-in controls that can be done during the step of library preparation that can provide absolute quantification of transcriptome and is reproducible when working with plasma samples (Everaert et al., 2019). Normalization can also be done based on the size of library and the length of the gene to avoid variability in results. Identification of biomarkers from raw data can be done through comparative analysis of the cell free RNA profiles or through machine learning methods. Comparative analysis provides simpler approach to identify genes and their expression associated to a phenotype. However, this can only determine the presence or absence of disease and prognosis. This methodology has been used to identify seven RNA signature that is associated with pre-eclampsia. However, the machine learning approach can infer more information related to the detection of biomarker signatures, prognosis, presence of disease and likelihood of a phenotype with better sensitivity and specificity. This approach has been used to identify a panel of 57 RNA biomarkers that can be used to detect COVID-19 with a precision of approximately 98.1% (Wang et al., 2022b).

## 8 Conclusion

The studies conducted in the field of liquid biopsies are limited. However, the application of liquid biopsies, especially ccf-mRNA biopsies is very diverse. The use of coding RNA molecules as promising biomarkers for diagnosis, prognosis, and therapeutic use holds potential in incorporating non-invasive diagnostic tests in routine clinical practice. The growing interest in RNAs as biomarkers has not been very recent, but the shift of focus from microRNA to long-coding RNAs has only been in last 5 years that has steered the discovery of several new disease-associated RNA. Even though there is much that needs to be explored and studied to successfully bring the use of mRNAs into clinical practice, the recent studies conducted on ccf-mRNA as biomarkers for various cancers and other diseases suggest that the use of ccf-RNA may complement ctDNA in bringing a new revolution for screening, diagnosis, disease monitoring and improve patient management.
